# Letters from Europe: The Great Hospitals: The Congress, Etc.

**Published:** 1890-09

**Authors:** E. J. Beall

**Affiliations:** Fort Worth


### Correspondence.

### LETTERS FROM EUROPE.

DRESDEN, August 2, 1890.

*Editor Daniel's Texas Medical Journal:*

From London I went to Paris; visited the Hotel Dieu, La Charitè, and the Hospital St. Louis. At the latter I saw Peau and witnessed five operations which he did. Peau is a sturdy, bold, bushy-headed Frenchman, and, as you know, has considerable reputation. I was surprised to learn that he is rather quachish; relies a good deal—like some Texas and other doctors—upon newspapers, fine clothing and electioneering indirectly in high places. He goes through a case with confidence and dispatch. Is very reserved in diagnosis, deferring positive opinion in three of the five cases I saw him in till the microscope should be consulted.

I saw nothing peculiar or different in his technique or dressings from that with which we are familiar. Patients, in many instances, after operation are wheeled out and an already anæsthetized one is placed upon the table. Thus expediting matters and enabling him to do this charity with consuming two days in each week. He ligates the larger vessels and the smaller ones are held by the clamp till the leisure moment comes for the assistants to apply permanent dressings outside the amphitheatre.

You will remember that the priority of the method of leaving the clamp forceps in place for hours after vaginal hysterectomies, has been controverted; usually this is considered a method of Peau.

I was not very much impressed with the personnel of Peau's assistants, nor the tidiness of persons and things around the operating table at the St. Louis hospital. I regretted that I could not stay long enough to see the work of the artistic surgeon Toudeux, also Doleris and Champonies, as well as Charcot; but as I had some difficulty in catching everything said in French—being of late years dull in that language—I could not afford to linger long in beautiful Paris and her renowned hospitals.

From Paris I passed Fontainbleu, Rouen and Lousana, and stopped at Geneva. At the latter place I was kindly received by the affable and accomplished Cordes and lady, and after carrying me to the surgical clinic and offering to take me through the maternity, of which he was head, I returned to the L'Angletere, and later was called for by the Doctor and Madame and wheeled to their country home; nine wiles away, to an excellent dinner, and back to the hotel at 10 p. m. His beautiful rural home is near the lake and we pass the summer house of a Rothchild, as well as a former home of Eugenie and Lola Montez in making the visit.

At Berne, the Swiss capital, I called and was kindly received by Dr. Sachs, Cocher's assistant, the latter being absent. The Cantonment hospital, over the surgical department of which Dr. Cocher presides, is an elegant and large hospital, and, as you know, the surgeon is able. His assistant was very kind, and I enjoyed very much the visit and the attendance at the clinic.

Though a day at Würzburg, I did not go to the hospital. This, you know, is the Bavarian home in which the clinicien Scanzoni lived and laid his reputation—a writer whom I have much enjoyed and hope received profit by the study of his writings.

This city, Dresden, you know, is the place of the great obstetric hospital, in which the great Winke (and antiseptic obsteritrician in advance of the times) lived and made fame ere he left for Munich. Leopold holds the place now. I intend spending the evening there.

To-morrow I will go to Berlin. The Congress meets Monday. Up to yesterday noon 4000 had registered. It is likely the number will reach 10,000.

The letters given me at New York will give me kind reception at the clinics of Bardeleben, Kuster and Bergmann. As I do not understand the German language I will remain only long enough to learn the technique of these men, when I shall return to London for a month of internal medicine, surgery, gynecolagy and eye.

Will write to you a special letter about the work at London.

Your friend, E. T. BEALL, M. D.

---

### INTERNATIONAL MEDICAL CONGRESS.

BERLIN, August, 1890.

*Editor Daniel's Texas Medical Journal:*

The work has I think been of high order, and the major part being presented by the Germans, who as would be expected, are largely in the majority. The attendance is about 7000, 500 of whom are Americans. I recognize nearly all those whom you and I meet at Washington. The entertainments are of high order and expensive, but those in this beautiful and wealthy city are well able for such expense. I write you no particulars, as by telegraph or journals you will have obtained particulars of the most important matters sooner than by course of mail.

I attended Bergmann's Clinic yesterday and Keester at the Augusta to-day; to-morrow will go to Bardalebenss, and next day with Prof. Prewitt of St. Louis, will see Hohn in laparotomy.

I observed Sir Jos. Lister, Sir William McCormac, and Mr. Peau yerterday at Bergmann's clinic. Bergmann's clinic was intended to display antiseptic treatment of wounds, but I saw nothing new, nothing but what we do in New York and Texas.

I wish to modify what I said in a former letter of Dr. Peau of Paris. Since writing you I have learned that Peau's having been beaten in the race for position in the Faculty at Paris was due more than anything else to political influences, rather than quackish practices, and though he is bold and at first sight looks ferocious, his appearance improves upon seeing him more frequently, and one gets to admiring the man. There is no question but that he has a cool steady hand and head, and has no fear of the knife. Perhaps my first informant was prejudiced against Peau and made a wrong impression upon me when I was in Paris and we were discussing the man after his clinic. He does an immense amount of work.

At a called meeting of the American members of the Congress at the Hotel Central, Unter den Linden, to-night, and from which I have just returned, it was decided not to present and urge an application from Chicago that the Congress of '93 meet in that city. It was thought that for us to urge the measure would not be doing justice to Russia, or would, perhaps, thwart the disposition many had of favoring Paris, with hope of bringing about a reconciliation of feeling between the profession of Germany and France, growing out of the late war between their countries. I hope and think Paris will get the Congress of '93. Very few French are attending the Congress at its present session.

Texas has a fair show of the American delegation.

I noticed some matters at the Augusta hospital that I would like to write about; one little measure I noticed was in attaching a bar with plaster below the heel, to prevent outward rotation when using Buck's extension for fracture; another was in placing the limb in position that the tenor vag. femoris muscle and fascia was relaxed during the treatment. I would like to note the difference in treatment of tubuncular hip disease between Kusier and Bergmann and compare their treatment with Barker of London, but time and paper forbid. Your friend,

E. J. BEALL, M. D.

